# The effect of genetically modified platelet-derived growth factor-BB over-expressing mesenchymal stromal cells during core decompression for steroid-associated osteonecrosis of the femoral head in rabbits

**DOI:** 10.1186/s13287-021-02572-7

**Published:** 2021-09-15

**Authors:** Roberto Alfonso Guzman, Masahiro Maruyama, Seyedsina Moeinzadeh, Elaine Lui, Ning Zhang, Hunter W. Storaci, Kaysie Tam, Elijah Ejun Huang, Takeshi Utsunomiya, Claire Rhee, Qi Gao, Zhenyu Yao, Yunzhi Peter Yang, Stuart B. Goodman

**Affiliations:** 1 Department of Orthopaedic Surgery, Stanford University School of Medicine, 300 Pasteur Drive, Edwards R155, Stanford, CA 94305 USA; 2 Department of Material Science and Engineering, Stanford University School of Medicine, Stanford, CA USA; 3 Department of Bioengineering, Stanford University School of Medicine, Stanford, CA USA; 4 Department of Mechanical Engineering, Stanford University School of Medicine, Stanford, CA USA; 5 Department of Orthopaedic Surgery, Stanford University School of Medicine, 450 Broadway Street, Redwood City, CA 94063 USA

**Keywords:** Osteonecrosis, Core decompression, Femoral head, Hip, Regenerative medicine, Rabbit

## Abstract

**Background:**

Approximately one third of patients undergoing core decompression (CD) for early-stage osteonecrosis of the femoral head (ONFH) experience progression of the disease, and subsequently require total hip arthroplasty (THA). Thus, identifying adjunctive treatments to optimize bone regeneration during CD is an unmet clinical need. Platelet-derived growth factor (PDGF)-BB plays a central role in cell growth and differentiation. The aim of this study was to characterize mesenchymal stromal cells (MSCs) that were genetically modified to overexpress PDGF-BB (PDGF-BB-MSCs) in vitro and evaluate their therapeutic effect when injected into the bone tunnel at the time of CD in an in vivo rabbit model of steroid-associated ONFH.

**Methods:**

In vitro studies*:* Rabbit MSCs were transduced with a lentivirus vector carrying the human PDGF-BB gene under the control of either the cytomegalovirus (CMV) or phosphoglycerate (PGK) promoter. The proliferative rate, PDGF-BB expression level, and osteogenic differentiation capacity of unmodified MSCs, CMV-PDGF-BB-MSCs, and PGK-PDGF-BB-MSCs were assessed. In vivo studies: Twenty-four male New Zealand white rabbits received an intramuscular (IM) injection of methylprednisolone 20 mg/kg. Four weeks later, the rabbits were divided into four groups: the CD group, the hydrogel [HG, (a collagen-alginate mixture)] group, the MSC group, and the PGK-PDGF-BB-MSC group. Eight weeks later, the rabbits were sacrificed, their femurs were harvested, and microCT, mechanical testing, and histological analyses were performed.

**Results:**

In vitro studies*:* PGK-PDGF-BB-MSCs proliferated more rapidly than unmodified MSCs (*P* < 0.001) and CMV-PDGF-BB-MSCs (*P* < 0.05) at days 3 and 7. CMV-PDGF-BB-MSCs demonstrated greater PDGF-BB expression than PGK-PDGF-BB-MSCs (*P* < 0.01). However, PGK-PDGF-BB-MSCs exhibited greater alkaline phosphatase staining at 14 days (*P* < 0.01), and osteogenic differentiation at 28 days (*P* = 0.07) than CMV-PDGF-BB-MSCs. In vivo*:* The PGK-PDGF-BB-MSC group had a trend towards greater bone mineral density (BMD) than the CD group (*P* = 0.074). The PGK-PDGF-BB-MSC group demonstrated significantly lower numbers of empty lacunae (*P* < 0.001), greater osteoclast density (*P* < 0.01), and greater angiogenesis (*P* < 0.01) than the other treatment groups.

**Conclusion:**

The use of PGK-PDGF-BB-MSCs as an adjunctive treatment with CD may reduce progression of osteonecrosis and enhance bone regeneration and angiogenesis in the treatment of early-stage ONFH.

## Background

Osteonecrosis is a chronic disease that results from vascular disruption and leads to death of bone and marrow elements [1, 2]. Osteonecrosis of the femoral head (ONFH) has the potential to progress to collapse of the femoral head and subsequent osteoarthritis (OA) of the hip [1, 2]. In fact, ONFH has been estimated to account for about 10% of total hip arthroplasties (THA) performed annually in the USA and up to half of those performed in Korea and Taiwan [2–4]. Within the USA, there is an incidence of about 20,000–30,000 new cases of ONFH diagnosed annually; other reports have estimated the annual incidence of osteonecrosis in China to be between 75,000 and 150,000, and in Japan to be between 12,000 and 24,000 [2, 4]. Moreover, those affected by ONFH are typically young, active individuals between 20 and 40 years old [4]. Although the exact etiology and pathophysiology of ONFH is unknown, it has been well established that glucocorticoid use, increased alcohol intake, and chronic inflammation play important roles in the development of the disease [1, 5].

Core decompression (CD) has become a mainstay of surgical management for early-stage ONFH as it improves clinical and radiographic outcomes when compared to nonoperative treatment [1, 2, 6]. However, despite widespread use, CD has an estimated success rate of only 65% according to a recent meta-analysis; many of the failed cases subsequently undergo THA [1, 7]. In light of this shortcoming, many augmentative treatment modalities, such as use of a tantalum rod insertion, bone grafts, and cell-based therapy have been attempted in order to improve outcomes of CD [1, 2]. However, these combinatorial approaches have yielded mixed outcomes and new complications. Consequently, the optimal treatment for early-stage ONFH is still an unmet clinical need [8]. Ultimately, these protocols must prioritize bone healing and revascularization, along with enhancement of mechanical strength to improve outcomes.

Platelet-derived growth factor (PDGF)-BB is a potential adjunctive therapy for improving outcomes of early-stage ONFH treatment undergoing CD. PDGF-BB is a growth factor that stimulates many cellular growth and differentiation processes [9]. Secretion of PDGF-BB by preosteoclasts increases migration of mesenchymal stromal cells (MSCs) and endothelial progenitor cells (EPCs) through the PI3K/Akt/FAK pathway as well as differentiation of osteoblasts through the Sphk1/S1P pathway [10]. PDGF-BB improved bone formation and strength in a mouse model of osteoporosis [11]. Similarly, in a murine OA model, PDGF-BB secreted by preosteoclasts increased angiogenesis, osteogenesis, and nerve ingrowth [12]. The lack of PDGF-BB secretion by preosteoclasts has also been linked to decreased osteoclastogenesis and bisphosphonate-related osteonecrosis of the jaw (BRONJ) in rats treated with zolendronate, which could be reversed with local PDGF-BB supplementation [13, 14]. Finally, PDGF-BB demonstrates immunomodulatory effects and curtails the adverse effects of pro-inflammatory stimuli. For example, the harmful effects of interleukin-1-beta (IL-1β) on chondrocytes were mitigated by PDGF-BB supplementation, which reduced activation of nuclear factor kappa B (NF-κB) and limited cleavage of caspase-3, resulting in decreased apoptosis [15].

Our group recently demonstrated that murine MSCs that were genetically modified to overexpress PDGF-BB accelerated cellular proliferation and promoted greater osteogenesis and mineralization in vitro [16]. Given these findings, we hypothesized that the addition of MSCs genetically modified to overexpress PDGF-BB may improve the outcomes of CD in the treatment of ONFH. The aim of this study was to evaluate the proliferative and osteogenic potential of PDGF-BB-overexpressing rabbit MSCs (PDGF-BB-MSCs) in vitro and to characterize the therapeutic effect of injecting these modified cells into the surgically created bone tunnel at the time of CD in an in vivo rabbit model of steroid-associated ONFH.

## Materials and methods

### In vitro study

#### Establishment of genetically modified PDGF-BB over-expressing rabbit MSCs

Rabbit bone marrow-derived MSCs were purchased (Cyagen Biosciences, Santa Clara, CA) and expanded in growth medium (GM) containing α-Minimal Essential Medium (α-MEM, Thermo Fisher Scientific, Rockford, IL), supplemented 10% MSC-certified fetal bovine serum (FBS, Life Technologies, Pleasanton, CA), and 1% antibiotic and antimycotic solution (100 units of penicillin, 100 µg of streptomycin, and 0.25 µg of amphotericin B per mL, Life Technologies) until passage 5. Two distinct types of PDGF-BB-MSCs were generated by infecting MSCs with the lentiviral vector carrying the human PDGF-BB gene under the cytomegalovirus (CMV) and human phosphoglycerate kinase (PGK) promoter, respectively. Briefly, the pCDH-CMV-PDGF-BB-EF1a-copGFP plasmid was constructed by insertion of PDGF-BB coded genes into the control transfer plasmid, pCDH-CMV-MCS-EF1a-copGFP (System Biosciences, Palo Alto, CA), and the pCDH-PGK-PDGF-BB-EF1a-copGFP plasmid was constructed by replacing the CMV promoter in pCDH-CMV-PDGF-BB-EF1a-copGFP with the PGK promoter from pCDH-PGK (Addgene, Watertown, MA) [16]. The control lentivirus vectors or PDGF-BB-overexpressing lentivirus vectors were produced in human embryonic 293 T cells (ATCC, Manassas, VA) by co-transfecting with the control or PDGF-BB-overexpressing transfer plasmids, packaged plasmid (psPAX2), and enveloped plasmid (pMD2G VSVG) using a calcium phosphate transfection kit (Takara Bio USA, Inc., Mountain View, CA) with 25 mmol/L chloroquine. The virus was mixed in serum-free medium with 6 µg/mL of polybrene (Sigma Aldrich, St. Louis, MO) and was infected into rabbit MSCs with culture for 6 h at the multiplicity of infection (MOI) of 80 [17]. At 4 days after infection, the infected cells were confirmed as GFP positive cells under the fluorescence microscope. Unmodified MSCs, CMV-PDGF-BB-MSCs, and PGK-PDGF-BB-MSCs from passage 7 were stored in a liquid nitrogen tank and were subsequently used for the in vitro and in vivo experiments as described below.

#### Cell culture

After thawing, unmodified MSCs, CMV-PDGF-BB-MSCs, and PGK-PDGF-BB-MSCs were seeded in T75 flasks and cultured with GM for one day. The GM was then replaced with fresh GM and the cells were cultured for an additional three days. The cells were then washed with phosphate-buffered saline (PBS) three times, trypsinized, and reseeded into well plates. Each cell type’s proliferative capacity, PDGF-BB expression level, and capacity for osteogenic differentiation were subsequently assessed.

#### Cell proliferation assay and PDGF-BB expression verification

Unmodified MSCs, CMV-PDGF-BB-MSCs, and PGK-PDGF-BB-MSCs were cultured in GM and seeded at a density of 2 × 10^4^ cells/well onto 24-well plates. The samples, which included the supernatant and cells, were collected at one, three, and seven days. For the samples collected at seven days, GM was replaced with fresh GM at three days. After collecting the supernatants, each plate was then washed three times with PBS and 1 mL of DNase/RNase free water was subsequently added into each well. Cellular DNA was released by three freeze–thaw cycles and the quantity of dsDNA in each well was measured by performing a Quant-iT PicoGreen dsDNA Assay (Invitrogen, Carlsbad, CA) according to the manufacturer’s instructions. Fluorescence was measured at 480/520 nm wavelength using a plate reader (SpectraMax M2e Microplate Reader, Molecular Devices, San Jose, CA), and the amount of dsDNA was calculated.

To verify expression of PDGF-BB, the supernatant at three and seven days was analyzed. The concentration of PDGF-BB was measured using a human PDGF-BB ELISA kit (R&D Systems, Minneapolis, MN) according to the manufacturer’s instructions. Optical absorbance at 450 nm was measured using a plate reader and PDGF-BB expression level was calculated.

#### Osteogenic assessment

Unmodified MSCs, CMV-PDGF-BB-MSCs, and PGK-PDGF-BB-MSCs were seeded at a density of 2 × 10^4^ cells/well onto 24-well plates for alkaline phosphatase (ALP) staining at 14 days and Alizarin Red staining at 28 days. The cells were cultured in osteogenic medium (OM) containing α-Minimal Essential Medium (α-MEM, Thermo Fisher Scientific) with 10% MSC-certified FBS, 1% antibiotic and antimycotic solution, 10 mM β-glycerophosphate (MP Biomedicals, Irvine, CA), 50 mM 1-ascorbic acid (Sigma Aldrich), 100 mM Vitamin D3 (Sigma Aldrich), and 100 nM dexamethasone (Sigma Aldrich). The culture medium was replaced every three to four days.

For the ALP staining, the supernatant from each well was aspirated after 14 days of culture, the cells were washed twice with 0.9% normal saline, and then fixed with 1 mL of 4% paraformaldehyde (PFA) for 15 min. Thereafter, the cells were stained with 300 µL of an ALP substrate solution (1-StepTM NBT/BICP substrate Solution, Thermo Fisher Scientific), and allowed to incubate overnight at 37 °C. The cells were then washed twice with PBS and allowed to dry. Each well was then imaged using a BZ-X 810 digital microscope (Keyence, Osaka, Japan) at 20× magnification. To quantify the percentage of each well that was stained, the area of positive staining was divided by the total area of each well. Briefly, ImageJ 1.53e (NIH) was used by two independent evaluators to set a threshold for positive staining, a consensus for the final threshold was reached, and the same software was used to measure the area of positive staining and the total well area.

For the Alizarin Red staining, the cells were washed twice with PBS, fixed with 4% PFA for 15 min, and then stained with 300 µL of 40 mM pH 4.1–4.3 Alizarin Red S (Sigma-Aldrich) and allowed to shake at 30 RPM at room temperature for 20 min. Subsequently, the stain was aspirated, and each well was washed with 1 mL of PBS and allowed to shake at 30 RPM at room temperature for 5 min. The wash process was repeated twice more, after which each well was imaged at 20× magnification as above. The percentage of staining in each well was calculated using the same process as the ALP staining quantification described above.

In order to control for variable amounts of starting genetic material, all results, except those of the PDGF-BB ELISA, were normalized by the average amount of dsDNA present in each well for each cell type, after one day of culturing. The results of the day three and day seven PDGF-BB ELISA were normalized by the average amount of dsDNA present in each well for each cell type, after three and seven days of culturing, respectively.

### In vivo study

#### Fabrication of the MSC-laden hydrogel

In order to inject MSCs into the surgically created bone tunnel at the time of CD, unmodified MSCs and PGK-PDGF-BB-MSCs were encapsulated in a hydrogel (HG) prior to surgery, as previously described [18]. Briefly, sodium alginate (Pfaltz & Bauer Inc, Waterbury, CT) was dissolved in calcium-free DMEM (Thermo Fisher Scientific) at 1% (w/v) concentration, after which 8 µL of NaOH (1 N) was added to 1 mL of solution. This alginate solution was stored at 4 °C. One hundred mg of sterile CaSO_4_ was then mixed with 1 mL of sterile deionized (DI) water and vortexed for 5 min at room temperature. Separately, calcium-free DMEM was added to collagen stock solution which contained collagen type 1 from rat tail (Corning Inc, NY) at 1:1 volume ratio at 4 °C. Then, 20 µL of the vortexed CaSO_4_ suspension was added to 1 mL of the collagen solution. Afterwards, unmodified MSCs or PGK-PDGF-BB-MSCs were added to this collagen precursor solution at a density of 10 million cells/mL, and this mixture was then added to the alginate solution at a 1:1 volume ratio. A previous study demonstrated that this resulted in calcium-driven alginate crosslinking within 15 min and a resultant hydrogel that was injectable through commercially available needles and stable right after injection into the bone tunnel [18]. This finalized MSC-laden HG contained cells at a density of 5 million cells/mL and was stored on ice prior to injection. Previously, this HG-MSC system demonstrated greater than 90% cell viability, 6.2-fold increase in DNA, and greater than 90% protein release at 14 days after encapsulation [18].

#### Rabbit surgery

All animal experiments were approved and performed in accordance with our institution’s Animal Care and Use Committee guidelines. Twenty-four male mature New Zealand rabbits (West Oregon Rabbit Company, OR), five to six months of age and weighing from 4.0 to 4.5 kg, received a single, intramuscular injection of 20 mg/kg of methylprednisolone acetate (MPS: Depo-Medrol®, Pfizer Inc, NY) four weeks prior to surgery [19–22]. Rabbits were divided into four groups (*n* = 6 in each): (1) the CD group, (2) the HG group, (3) the MSC group, and (4) the PGK-PDGF-BB-MSC group. Note, the cells in groups 3 and 4 were encapsulated in HG identical to that used in group 2.

Prior to surgery, rabbits were anesthetized with xylazine (4 mg/kg) and ketamine (40 mg/kg) and inhaled isoflurane was provided for additional anesthesia. Buprenorphine SR (0.15 mg/kg) and enrofloxacin (25 mg/kg) injections were also given. For the CD group, a 20 mm skin incision was made over the left proximal lateral thigh, and the vastus lateralis muscle was reflected [21, 22]. A 2-mm-diameter round burr was used to create a small hole at the distal end of the third trochanter, and fluoroscopy was subsequently used to guide the insertion of a 0.9-mm guide wire from the hole to the center of the femoral head. Next, a cannulated drill bit was used to drill over the guide wire to create a 3-mm-diameter bone tunnel from the third trochanter to 2 mm distal to the surface of the femoral head. For the HG group, 200 µL of HG, prepared as described above, was injected into the bone tunnel prior to wound closure. For the MSC and PGK-PDGF-BB-MSC groups, 1 million unmodified MSCs or PGK-PDGF-BB-MSCs encapsulated in 200 µL of HG were injected into the bone tunnel prior to wound closure. The wound was closed with non-absorbable suture and antibiotics were administered for two days, postoperatively. Rabbits were kept in cages and allowed free activities until eight weeks after surgery, at which point they were euthanized, and the femurs were harvested.

#### MicroCT analysis

A microCT (SkyScan 1276 micro-CT system, Bruker, Kontich, Belgium) with 20 µm resolution at 2016 × 1344, AI 1 mm, 85 kV, 200 µA, with two average frames at every 0.4° angle step was used to scan the proximal femurs. NRecon software (version 1.6) and GEMS MicroView software (eXplore MicroView v.2.5, Analysis Plus, GE Healthcare, Toronto, Canada) were used for data reconstruction and analysis, respectively. Bone mineral density (BMD, mg/mm^3^) and bone volume fraction (BVF), inside and outside the CD area in the femoral head, were calculated. Briefly, a cylindrical region of interest (ROI) with an 11-mm diameter × 6-mm length was positioned to cover the entire femoral head, and a second cylindrical ROI with a 3-mm diameter was co-centrally positioned inside the CD area at the bottom of the 6-mm thickness of the femoral head. The total volume (TV, mm^3^), bone volume (BV, mm^3^), and bone mineral content (BMC, mg) within each region were measured. The area outside the CD was defined as the entire femoral head excluding the area within the CD, and BMD and BVF were calculated using the data for the whole femoral head minus the data within the CD. A threshold of bony tissue was determined by a phantom. Specimens were then stored in a − 80 °C freezer prior to subsequent biomechanical testing.

#### Biomechanical testing

A Materials Testing System fitted with a 2 kN load cell (5944 Instron Corporation, Norwood, CA) was used for mechanical testing. Specimens were thawed and kept moist with PBS throughout testing at room temperature.

First, the stiffness of the femoral head surface was calculated by performing an indentation test [21, 22]. Specimens were transversely cut 60 mm distal to the femoral head, and the distal end was potted with polymethylmethacrylate (PMMA). Additional PMMA was packed under the inferior surface of the femoral neck to avoid bone deflection/fracture while testing. Specimens were secured in a swivel vise with the long axis of the femur placed 17° from the vertical in the coronal plane, resulting in a 17° laterally directed load vector on the femoral head. Although not physiologic in rabbits, this orientation was chosen to approximate the frontal plane angle at peak load during a variety of daily activities in humans [23]. A 1.6 mm-diameter indenter was aligned with the Ligamentum Teres in the sagittal plane and used to indent the surface of the femoral head. A 1 N compressive preload was applied, and the specimens were subsequently loaded at a displacement rate of 10 mm/min until 0.5 mm of displacement or 300 N of load were reached. Load and displacement data were recorded at 100 Hz, and stiffness of the femoral head surface was calculated from the linear portion of the load vs. displacement curve.

Next, the stiffness of the central portion of the femoral head was calculated by performing another indentation test [21, 22]. The femoral head was cut into two 4-mm-thick segments using a 0.5-mm-hand saw with a hand-made saw guide set perpendicular to the bone tunnel axis. The proximal segment was stored in PFA (pH 7.4) for subsequent histological analysis, while the middle fragment was used for the indentation test. A 2.3-mm-diameter indenter was positioned to indent the fragment through the axis of the bone tunnel. Again, a 1 N compressive preload was applied, and the specimens were then loaded at 10 mm/min until failure was observed. Data recording and stiffness calculation were completed the same way as the first indentation test described above.

#### Calculation of empty lacunae outside of the CD area

**Fig. 1:**
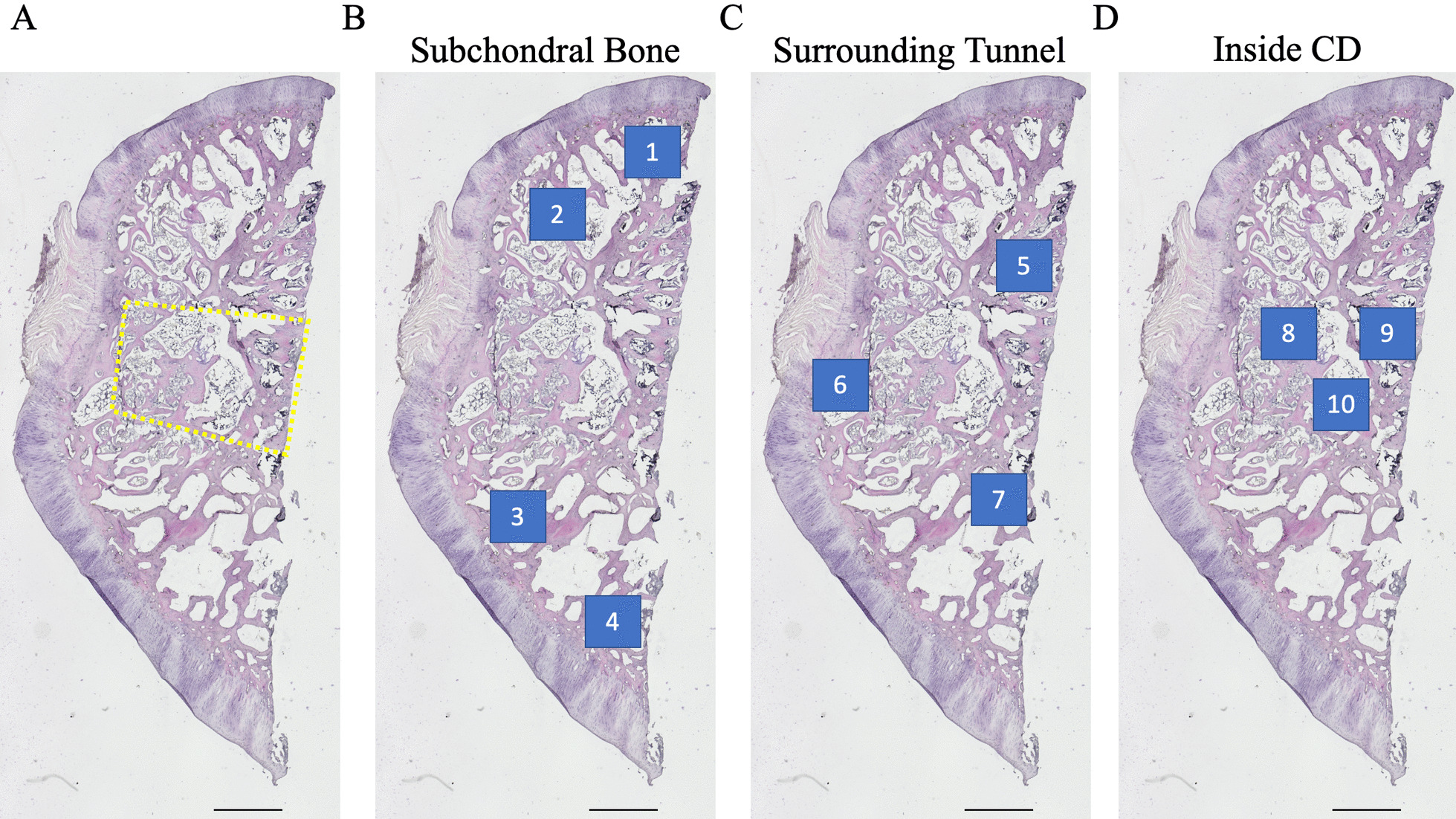
Methods for histology field selection. Histology slides of the femoral head were imaged using a Keyence light microscope at ×40 magnification to visualize the entirety of each specimen. **a** Representative image of an H&E stained specimen with the borders of the CD area outlined with a dashed yellow quadrilateral. **b** The same image is used to demonstrate the position of the four fields that were used to image the subchondral bone at ×200 magnification for H&E, TRAP, and CD31 stained sections. **c** The same image is used to demonstrate the position of the three fields that were used to image the bone surrounding the bone tunnel at ×200 magnification for H&E, TRAP, and CD31 stained sections. **d** The same image is used to demonstrate the position of the three fields that were used to image the newly formed bone within the bone tunnel at ×200 magnification for the CD31 stained sections. Scale bar: 1000 µm. *H&E* hematoxylin and eosin, *CD*, core decompression, *TRAP* tartrate-resistant acid phosphatase, *CD31* cluster of differentiation 31

The proximal segment of the femoral head was decalcified with 0.5 M ethylenediaminetetraacetic acid (EDTA, pH 7.4) and subsequently embedded in optimal cutting temperature (OCT) compound to obtain 8-µm-thick longitudinal frozen sections collinear with the long axis of the bone tunnel. After hematoxylin and eosin (H&E) staining, seven consistent fields outside of the CD area, four subchondral fields and three fields bordering the edge of the bone tunnel, were imaged at 200× magnification using a BZ-X 810 digital microscope (Keyence) (Fig. 1a–c). The tissue was considered to contain osteonecrotic lesions when the following two requirements were satisfied: (1) empty lacunae and pyknotic nuclei of osteocytes within the bone, (2) bone marrow cell necrosis, fatty bone marrow without hematopoietic cells, scant bone marrow, or reparative tissue such as accumulating multinuclear cells, granulation tissue, fibrosis, or appositional bone formation with osteoblast-like cells around the osteonecrotic lesion within the region of bone marrow [19, 22, 24]. To further quantify the degree of osteonecrosis, a deep convolutional neural network was utilized to classify cells in histological sections to determine the percentage of empty lacunae. Briefly, a model was developed to classify objects into one of two classes: osteocyte or empty lacunae. A total of *N* = 135 H&E images were manually counted by two independent and blinded evaluators to establish the ground truth. Of this data, *N* = 27 images were randomly selected and segmented into 3 × 3 matrices for a total of *n* = 243 sub-images. The sub-images were then split into training and validation sets according to the Pareto principle (80–20 rule). Each object within the sub-images in the training and validation set was annotated using the ground truth labels. The classifier was then trained via supervised learning using the Faster RCNN framework and Inception-V2 model, which were downloaded directly from the Tensorflow Object Detection repository. Training ended when the reported loss value, the cumulative measure of error between predicted and observed outputs, consistently fell below 0.05. The accuracy of the model was then validated by comparing the model predictions for new images with the results from manual counting (the ground truth). After confirming a high correlation between the predictive results and ground truth (adjusted *R*^2^ = 0.96), the percentage of empty lacunae in the remaining H&E stained images was evaluated using the cell classifier.

#### Histological assessment of osteoclastogenesis and angiogenesis

Frozen sections of the proximal femoral head fragment were also stained to detect tartrate-resistant acid phosphatase (TRAP) and CD31 (Platelet Endothelial Cell Adhesion Molecule-One; PECAM-1). The TRAP histochemical staining kit (Sigma Aldrich) was used according to the manufacturer’s instructions. For CD31 staining, sections were treated with proteinase K (S3020, Dako Cytomation, CA) and endogenous peroxidase activity was blocked with 0.3% hydrogen peroxide treatment. A mouse monoclonal anti-rabbit CD31 antibody (diluted 1:800, NB600-562; Novus Biologicals, Littleton, CO) was used as a primary antibody. A secondary antibody (Histofine Simple Stain MAX-PO (M), 414132F, Nichirei Bioscience, Tokyo, Japan) and 3,3′-diaminobenzidine (DAB) solution (34002, Thermo Fisher Scientific) were used to visualize the bound primary antibody. The TRAP and CD31 stained sections were then counterstained with hematoxylin.

**Fig. 2:**
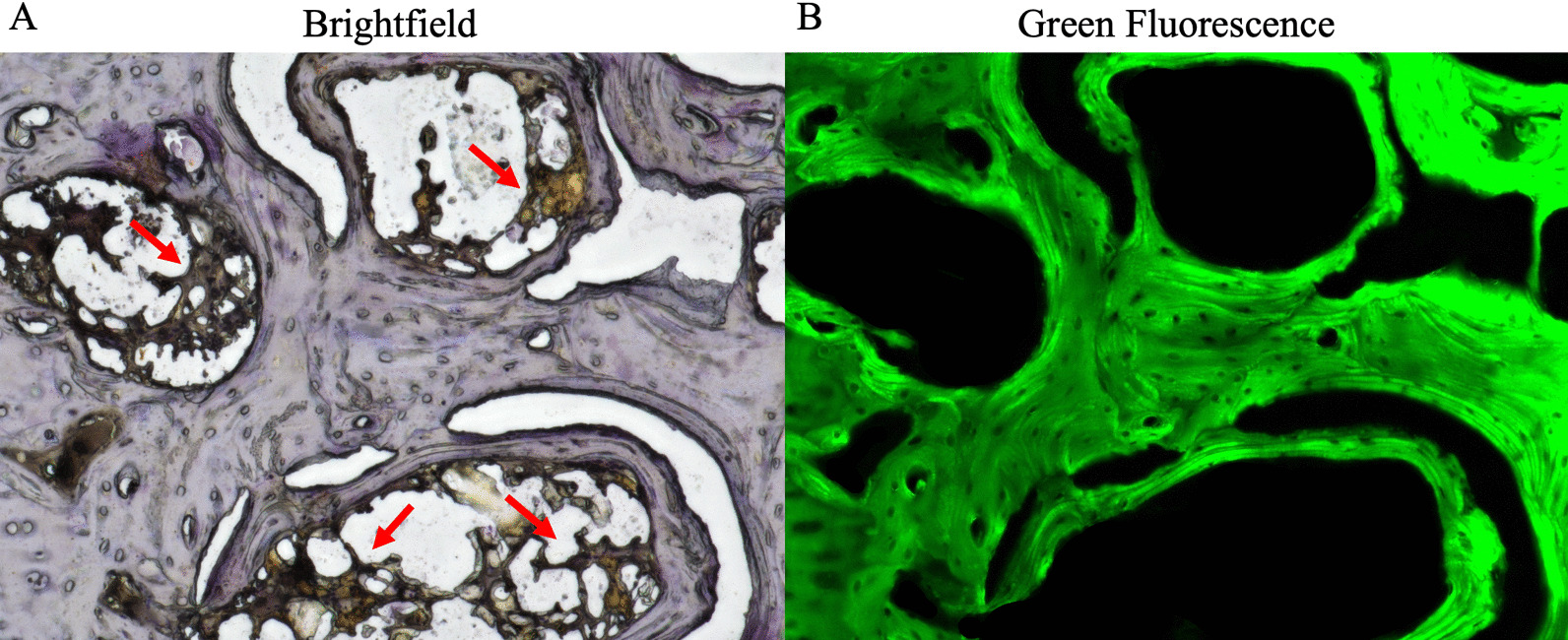
Methods for osteoclastogenesis evaluation. Femoral head specimens were stained for TRAP. **a** Representative image of TRAP stained specimen at ×200 magnification using a brightfield light. The number of osteoclasts (red arrows) in each brightfield image was manually counted by two blinded evaluators. **b** The same field was imaged using green fluorescent light in order to specifically image the bone. The area of bone was then calculated using imageJ software and osteoclast density was calculated as number of osteoclasts divided by bone area (#/mm2). *TRAP* tartrate-resistant acid phosphatase

The same seven fields that were imaged in the H&E stained sections were selected in each of the TRAP stained sections and captured at 200× magnification (Fig. 1b, c). For CD31 stained sections, in addition to the seven aforementioned fields, three fields from within the region of the bone tunnel were imaged at 200× and analyzed in order to assess the angiogenic effects of the treatment groups on the newly formed bone (Fig. 1b–d). ImageJ was used to quantitatively assess the images as follows: For TRAP staining, the number of osteoclasts in each image was counted by two independent, blinded evaluators. The total bone area for each image was then measured by imaging each field at 200× with green fluorescence light (Fig. 2) in accordance with prior methods, and the number of osteoclasts per area of bone was subsequently calculated for each image [25]. For CD31 staining, the number of CD31-positive stained microvessels in each image was also counted by two blinded evaluators. In order to ensure objective manual counting, each image was assigned a randomized number so that the evaluators would be blinded to the treatment group corresponding to each image. Note: one section from each rabbit sample was used for each of the three stains performed (e.g., 24 sections underwent H&E, TRAP, and CD31+ staining for a total of 72 stained sections).

#### Statistical analysis

Data are reported as mean +/− standard deviation. All of the in vitro experiments were completed in triplicate. An unpaired t test was used for comparison of PDGF-BB expression level between CMV-PDGF-BB-MSCs and PGK-PDGF-BB-MSCs. A one-way ANOVA with post hoc Tukey test was used for the remaining in vitro studies, microCT analysis, and mechanical testing. A multivariable mixed-effects model was used for the histological analyses, in which treatment group and femoral head position served as fixed effects in order to also consider how the relative position within the femoral head of each image may influence the result. To evaluate the accuracy of manual counting the internal reliability of the number of TRAP-positive osteoclasts and number of CD31-positive microvessels between the two investigators was assessed by intraclass correlation coefficients (ICC). Statistical significance was set as *P* < 0.05. The statistical analysis was conducted using Prism 9.0.0 (GraphPad Software) and RStudio version 1.2.5402. Power analysis using previous data indicated that a sample size of six per group would provide 80% statistical power to detect significant differences among the groups (*α* = 0.05, *β* = 0.20) [21].

A prior study from our group utilized samples from the first three treatment groups (i.e., the CD, HG, and MSC groups) in the current study for a separate set of analyses [26]. However, the current study has expanded the methods used for evaluation in two important ways. Firstly, the current study employed a novel deep learning algorithm to measure the percentage of empty lacunae in each of the H&E stained sections, and thus allowed for a consistent, unbiased evaluation of the histologic evidence of osteonecrosis. Secondly, each histologic section was assessed by dividing the image into two separate fields: the subchondral bone area, i.e., the area immediately beneath the articular cartilage where the early changes of osteonecrosis are typically first noted; and the bone surrounding the bone tunnel within the femoral head, adjacent to the site where the cells were injected.

## Results

### In vitro study

#### The PGK promoter led to more accelerated cellular proliferation than the CMV promoter

**Fig. 3:**
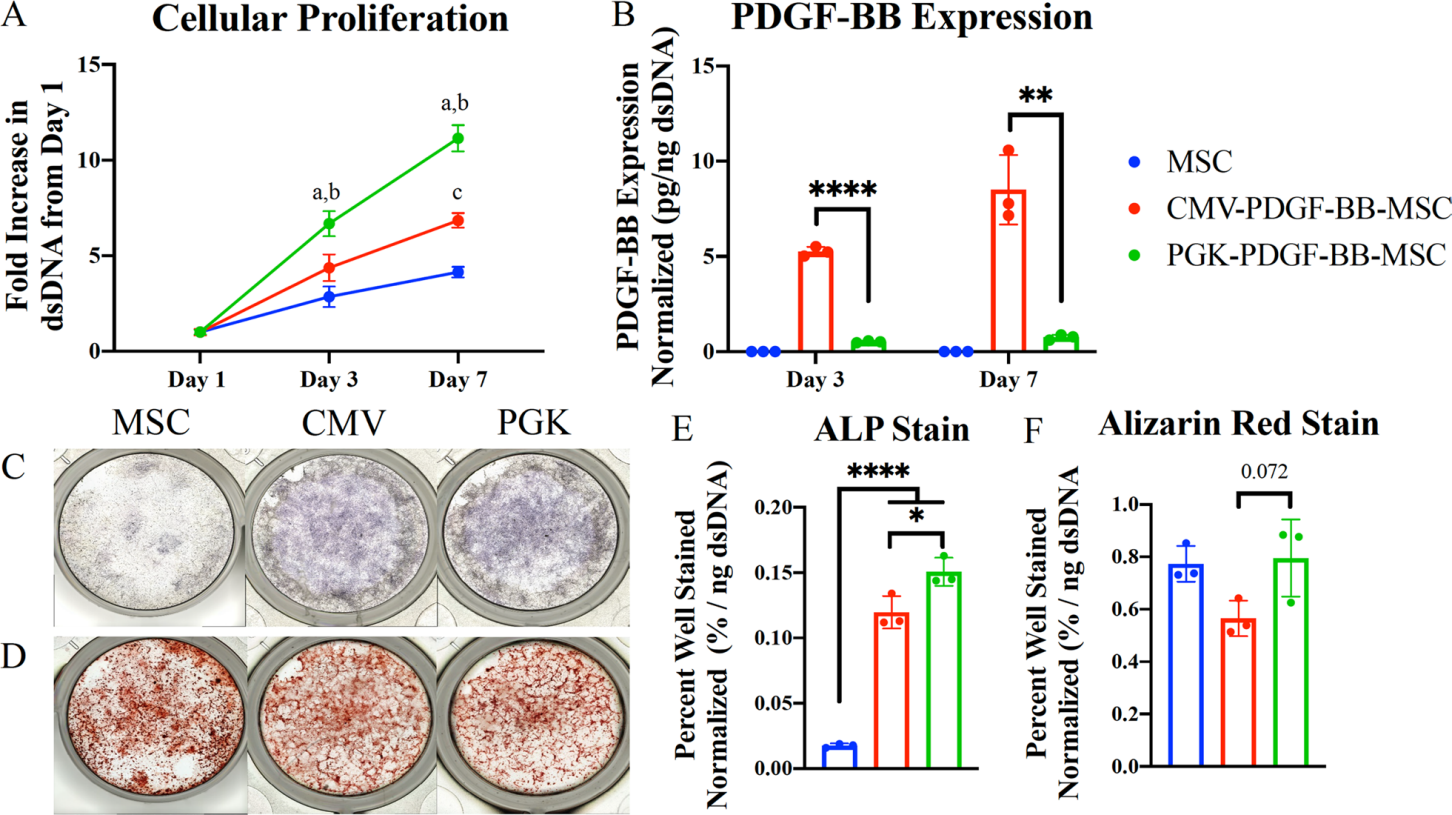
In vitro characterization of MSCs. **a** The proliferation rate of each cell type was evaluated by measuring the fold increase in dsDNA from day one, according to the PicoGreen dsDNA quantitation assay results. After three days of culturing, the PGK-PDGF-BB-MSCs demonstrated significantly greater proliferation than the unmodified MSCs (*P* < 0.001) and the CMV-PDGF-BB-MSCs (*P* < 0.05). After seven days of culturing, the PGK-PDGF-BB-MSCs demonstrated significantly greater proliferation than the unmodified MSCs and CMV-PDGF-BB-MSCs (*P* < 0.0001 for both). The CMV-PDGF-BB-MSCs also demonstrated greater proliferation than the unmodified MSCs (*P* < 0.01). **b** Expression level of PDGF-BB was measured by ELISA and normalized by the amount of dsDNA present for each cell type. Expression of PDGF-BB by the unmodified MSCs was undetectable. After three and seven days of culturing, the CMV-PDGF-BB-MSCs exhibited significantly greater expression of PDGF-BB than the PGK-PDGF-BB-MSCs (*P* < 0.0001 and *P* < 0.01, respectively). Osteogenic differentiation capacity of each cell type was assessed by staining for (**c**, **e**) ALP and with (**d**, **f**) Alizarin Red and normalizing by the amount of dsDNA that was present for each cell type on day one. After 14 days of culturing, PGK-PDGF-BB-MSCs demonstrated greater staining for ALP than unmodified MSCs (*P* < 0.0001) and CMV-PDGF-BB-MSCs (*P* < 0.05). Additionally, CMV-PDGF-BB-MSCs demonstrated greater staining than the unmodified MSCs (*P* < 0.0001). After 28 days of culturing, PGK-PDGF-BB-MSCs demonstrated a trend towards greater staining than the CMV-PDGF-BB-MSCs (*P* = 0.072). *PDGF-BB* platelet-derived growth factor-BB, *MSCs* mesenchymal stromal cells, *CMV-PDGF-BB-MSCs* genetically modified cytomegalovirus promoter PDGF-BB over-expressing MSCs, *PGK-PDGF-BB-MSCs* genetically modified phosphoglycerate kinase promoter PDGF-BB over-expressing MSCs; *dsDNA* double-stranded DNA, *ELISA* enzyme linked immunosorbent assay, *ALP* alkaline phosphatase. *P < 0.05, **P < 0.01, ***P < 0.001, ****P < 0.0001

The results of the PicoGreen Assay used to assess the proliferative rate after three and seven days of culturing are shown in Fig. 3a. At three days, the quantity of dsDNA in the PGK-PDGF-BB-MSCs was 6.67 +/− 0.65 fold greater than at day one, which was significantly greater than both the CMV-PDGF-BB-MSCs (4.37 +/− 0.70, *P* < 0.05) and unmodified MSCs (2.86 +/− 0.53, *P* < 0.001). At seven days, the quantity of dsDNA in the PGK-PDGF-BB-MSCs was 11.14 +/− 0.69 fold greater than at day one, which was significantly greater than both the CMV-PDGF-BB-MSCs (6.85 +/− 0.39, *P* < 0.0001) and unmodified MSCs (4.14 +/− 0.28, *P* < 0.0001). Furthermore, the increase in dsDNA in the CMV-PDGF-BB-MSCs trended towards being greater than unmodified MSCs after three days of culturing (*P* = 0.058) and was significantly greater after seven days of culturing (*P* < 0.01).

#### The CMV promoter led to greater expression of PDGF-BB than the PGK promoter

The results of expression level of PDGF-BB by ELISA at three and seven days are shown in Fig. 3b. The expression of PDGF-BB in the unmodified MSCs was undetectable. Conversely, the expression of PDGF-BB in the CMV-PDGF-BB-MSCs was significantly greater than the PGK-PDGF-BB-MSCs at three days (5.25 +/− 0.25 vs. 0.52 +/− 0.05 pg/ng dsDNA, *P* < 0.0001) and seven days (8.51 +/− 1.83 vs. 0.76 +/− 0.14 pg/ng dsDNA, *P* < 0.01).

#### PDGF-BB enhances ALP expression but not mineralization

The presence of ALP in each well was quantified by staining the enzyme after 14 days of culturing, as is seen in Fig. 3c, e. PGK-PDGF-BB-MSCs (0.145 +/− 0.005%/ng dsDNA) resulted in a greater percentage of staining than CMV-PDGF-BB-MSCs (0.116 +/− 0.012%/ng dsDNA, *P* < 0.01) and unmodified MSCs (0.019 +/− 0.003%/ng dsDNA, *P* < 0.0001). Furthermore, CMV-PDGF-BB-MSCs resulted in greater staining than unmodified MSCs (*P* < 0.0001).

The results of Alizarin Red staining at 28 days are shown in Fig. 3d, f. PGK-PDGF-BB-MSCs (0.795 +/− 0.147%/ng dsDNA) trended towards increased staining than CMV-PDGF-BB-MSCs (0.565 +/− 0.068%/ng dsDNA, *P* = 0.072), but was not significantly different than unmodified MSCs (0.773 +/− 0.068%/ng dsDNA). Moreover, no significant differences were detected between CMV-PDGF-BB-MSCs and unmodified MSCs.

### In vivo study

#### PDGF-BB-overexpressing MSCs enhanced mineralization outside of the CD area

**Fig. 4:**
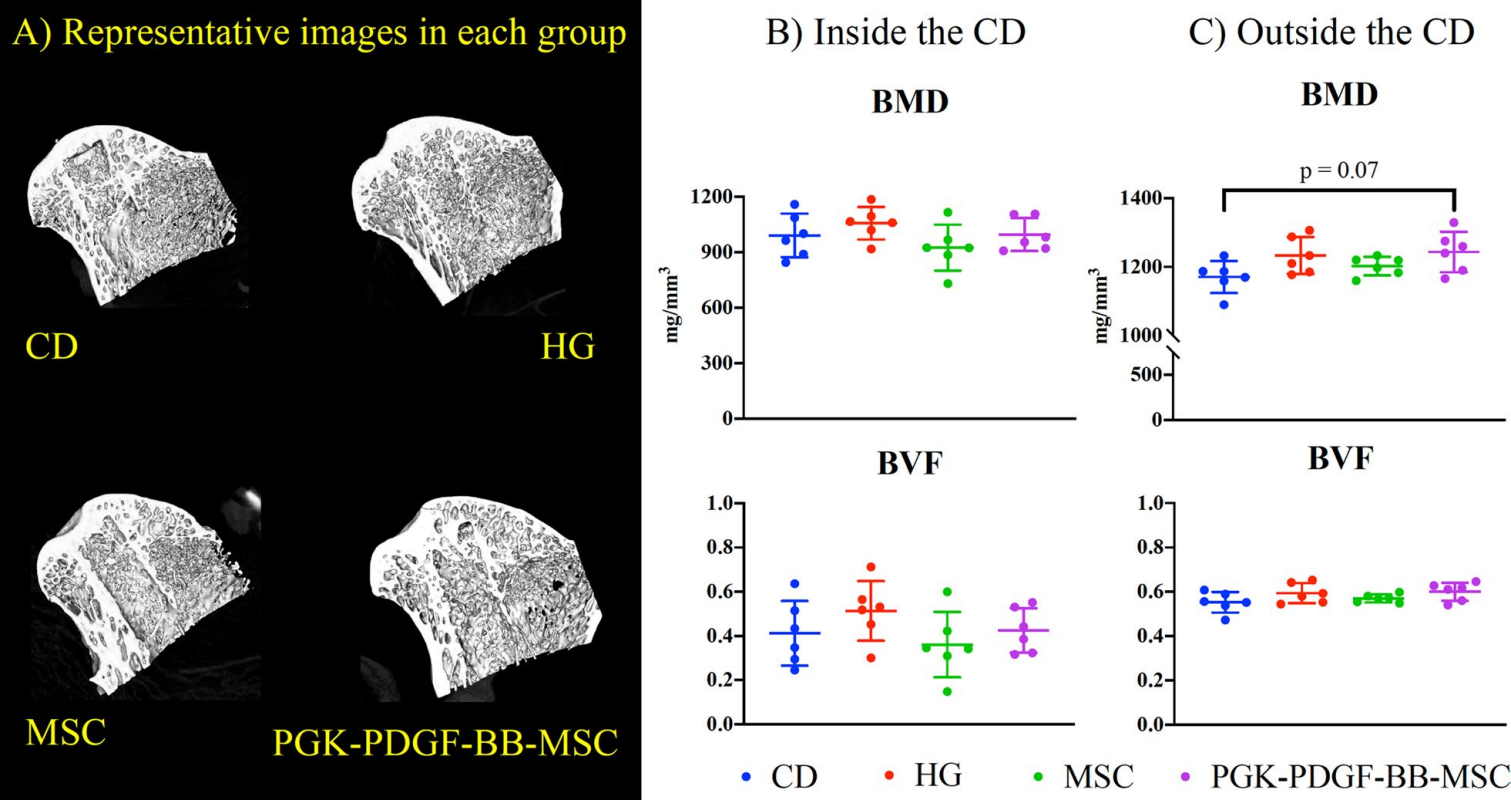
MicroCT analysis. BVF and BMD were assessed using micro-CT. **a** Representative images of the femoral head for each treatment group are shown. **b** Within the CD area, no significant differences were observed between treatment groups. **c** Outside of the CD area, the PGK-PDGF-BB-MSC group demonstrated a trend towards greater BMD than the CD group (*P* = 0.07). No differences were noted between treatment groups with regards to BVF. *CD* core decompression, *HG* hydrogel, *MSC* mesenchymal stromal cell, *PGK-PDGF-BB-MSC* genetically modified phosphoglycerate kinase promoter platelet-derived growth factor-BB over-expressing MSC, *BMD* bone mineral density, *BVF* bone volume fraction

The results of the microCT analysis are shown in Fig. 4. The rabbits in the PGK-PDGF-BB-MSC group (1243 +/− 58.9 mg/mm^3^) had a trend towards greater BMD in the area of the femoral head outside of the bone tunnel compared to the CD group (1171 +/− 46.9, *P* = 0.074), but there were no significant differences when comparing with the HG group (1233 +/− 53.4) or MSC group (1202 +/− 27.3). Additionally, no other significant differences were detected between any of the treatment groups with regards to BMD or BVF, inside or outside of the bone tunnel area.

#### No significant differences in bone stiffness were detected

**Fig. 5:**
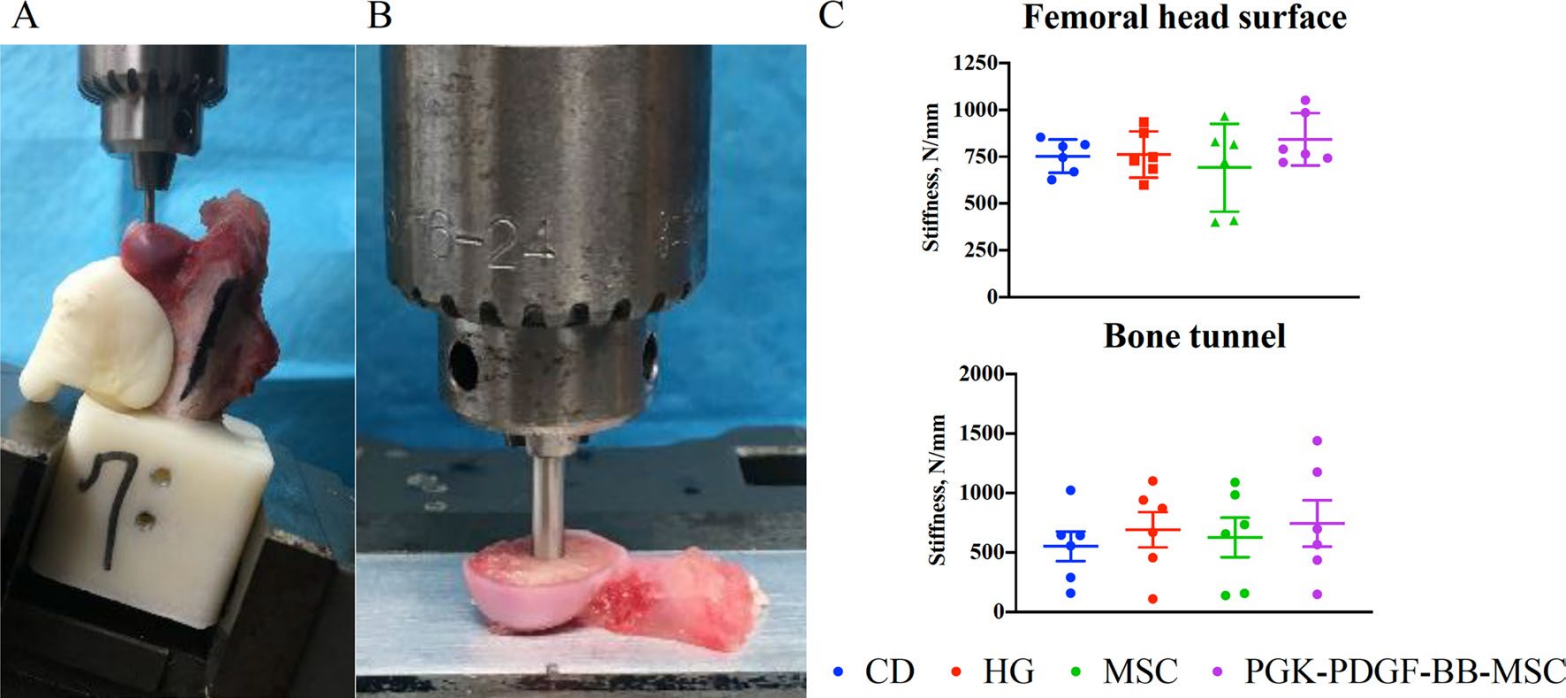
Mechanical testing. **a**, **b** Testing setup for the indentation tests of the femoral head surface and bone tunnel, respectively. **c** No differences were observed between treatment groups with regards to the stiffness of the femoral head surface or newly formed bone in the region of the bone tunnel. *CD* core decompression, *HG* hydrogel, *MSC* mesenchymal stromal cell, *PGK-PDGF-BB-MSC* genetically modified phosphoglycerate kinase promoter platelet-derived growth factor-BB over-expressing MSC

The results of the mechanical testing are shown in Fig. 5. Although the PGK-PDGF-BB-MSC group had a greater absolute femoral head and bone tunnel stiffness than the other three groups, these differences did not reach statistical significance. Likewise, there were no significant differences between any of the remaining treatment groups.

#### Augmentation of CD with PDGF-BB-overexpressing MSCs led to diminished histological evidence of osteonecrosis

**Fig. 6:**
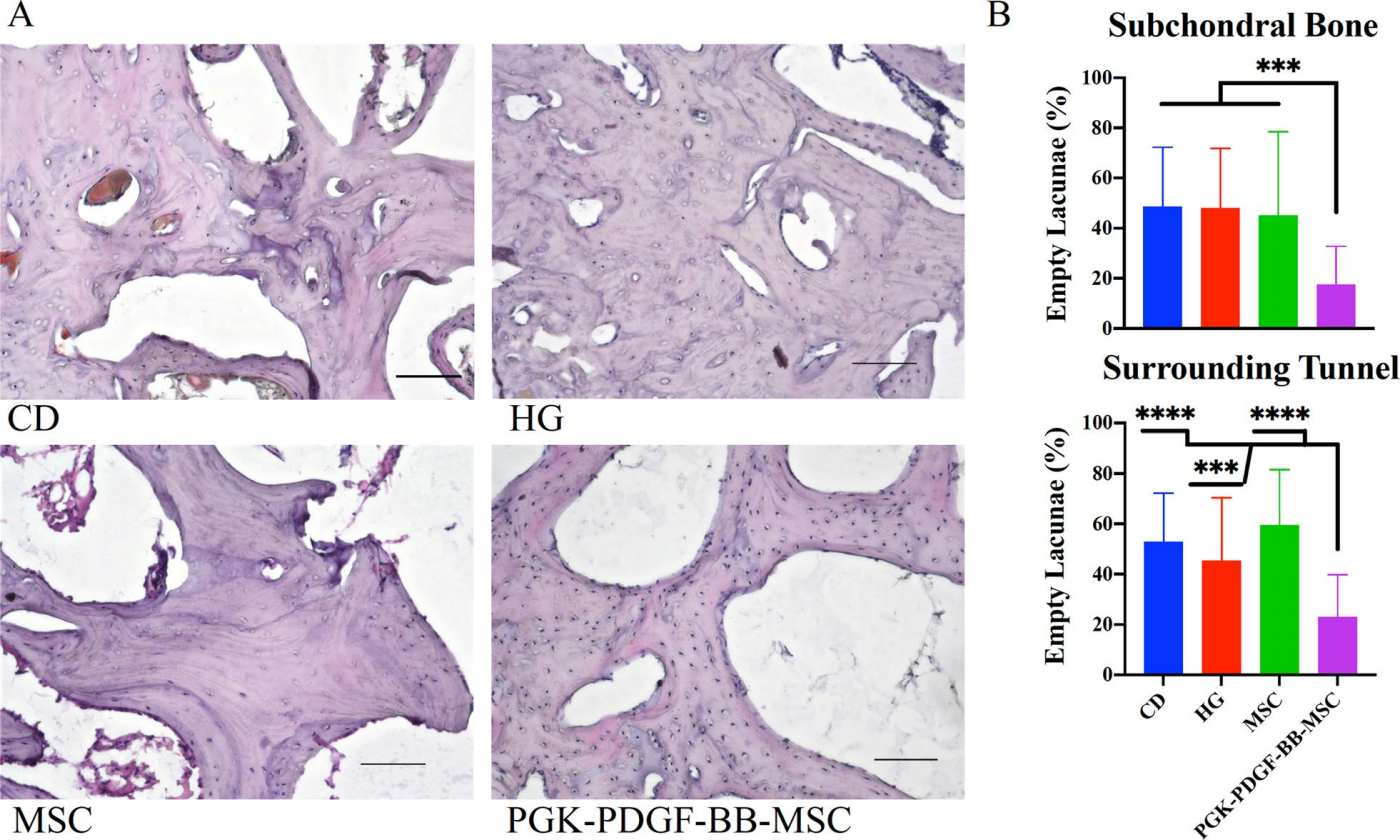
Assessment of osteonecrotic lesions. H&E staining was performed to evaluate the extent of osteonecrotic lesions within the femoral head. **a** Representative images of each treatment group are shown. Empty lacunae and pyknotic cells were observed in all treatment groups. Additionally, degenerative changes of the bone marrow such as granulation tissue were observed, indicating that osteonecrosis had taken place in all groups. **b** Within the subchondral bone and in the bone surrounding the bone tunnel area, the PGK-PDGF-BB-MSC group demonstrated a significantly lower percentage of empty lacunae than the other treatment groups (*P* < 0.001 for all). Scale bar: 100 µm. H&E, hematoxylin and eosin; *CD* core decompression, *HG* hydrogel, *MSC* mesenchymal stromal cell, *PGK-PDGF-BB-MSC* genetically modified phosphoglycerate kinase promoter platelet-derived growth factor-BB over-expressing MSC. ***P < 0.001, ****P < 0.0001

Representative images of the H&E staining in the femoral head are shown in Fig. 6. Empty lacunae and pyknotic osteocytes were observed in all groups, indicating the presence of osteonecrotic lesions within the femoral head of all rabbits. The results of the quantitative analysis of empty lacunae are shown in Table 1. When evaluating the subchondral bone, the PGK-PDGF-BB-MSC group demonstrated significantly lower percentage of empty lacunae (*P* < 0.001 for all) than the CD, the HG, and the MSC groups. Similarly, analysis of the bone immediately surrounding the surgically created bone tunnel showed that the PGK-PDGF-BB-MSC group resulted in significantly lower percentage of empty lacunae than the CD (*P* < 0.0001), the HG (*P* < 0.001), and the MSC group (*P* < 0.0001). However, no significant differences were observed between any of the other treatment groups in either region of the femoral head.

**Table 1:** Results of the evaluation of osteonecrosis

Average percentage (%) of empty lacunae by position in femoral head (SD)						
	Subchondral bone					
	1	2	3	4	Total	*P* value
CD	41.4 (28.7)	51.2 (22.6)	65.8 (16.7)	35.6 (19.2)	48.5 (23.8)	< 0.001
HG	46.6 (22.6)	65.8 (19.9)	50.9 (20.9)	28.8 (20.3)	48.0 (23.7)	< 0.001
MSC	58.9 (31.7)	71.6 (16.7)	40.7 (34.2)	8.8 (3.1)	45.0 (33.4)	< 0.001
PGK-PDGF-BB-MSC	16.1 (15.7)	12.0 (9.9)	19.1 (12.5)	23.0 (22.2)	17.5 (15.3)	1.0

	Surrounding tunnel				
	5	6	7	Total	*P* value
CD	59.8 (23.1)	52.8 (22.1)	46.2 (11.5)	52.9 (19.3)	< 0.0001
HG	64.2 (22.8)	44.9 (24.9)	27.1 (13.2)	45.4 (25.1)	< 0.001
MSC	72.6 (13.4)	55.7 (21.9)	50.4 (25.7)	59.6 (21.9)	< 0.0001
PGK-PDGF-BB-MSC	30.3 (20.7)	24.4 (17.6)	14.0 (7.8)	22.9 (16.8)	1.0

The average percentage of empty lacunae for each position in the femoral head as described in Fig. 1 was calculated for each treatment group. The average percentage of empty lacunae for each region in the femoral head was calculated for each treatment group. A mixed effects model, where treatment group and femoral head position served as fixed effects, was used to perform the statistical analysis. The *P* values shown correspond to comparisons with the PGK-PDGF-BB-MSC treatment group. Comparisons between all other treatment groups did not reach statistical significance. *SD* standard deviation, *CD* core decompression, *HG* hydrogel, *MSC* mesenchymal stromal cell, *PGK-PDGF-BB-MSC* genetically modified phosphoglycerate kinase promoter platelet-derived growth factor-BB over-expressing MSC

#### PDGF-BB-overexpressing MSCs accelerated osteoclastogenesis in a localized fashion

**Fig. 7:**
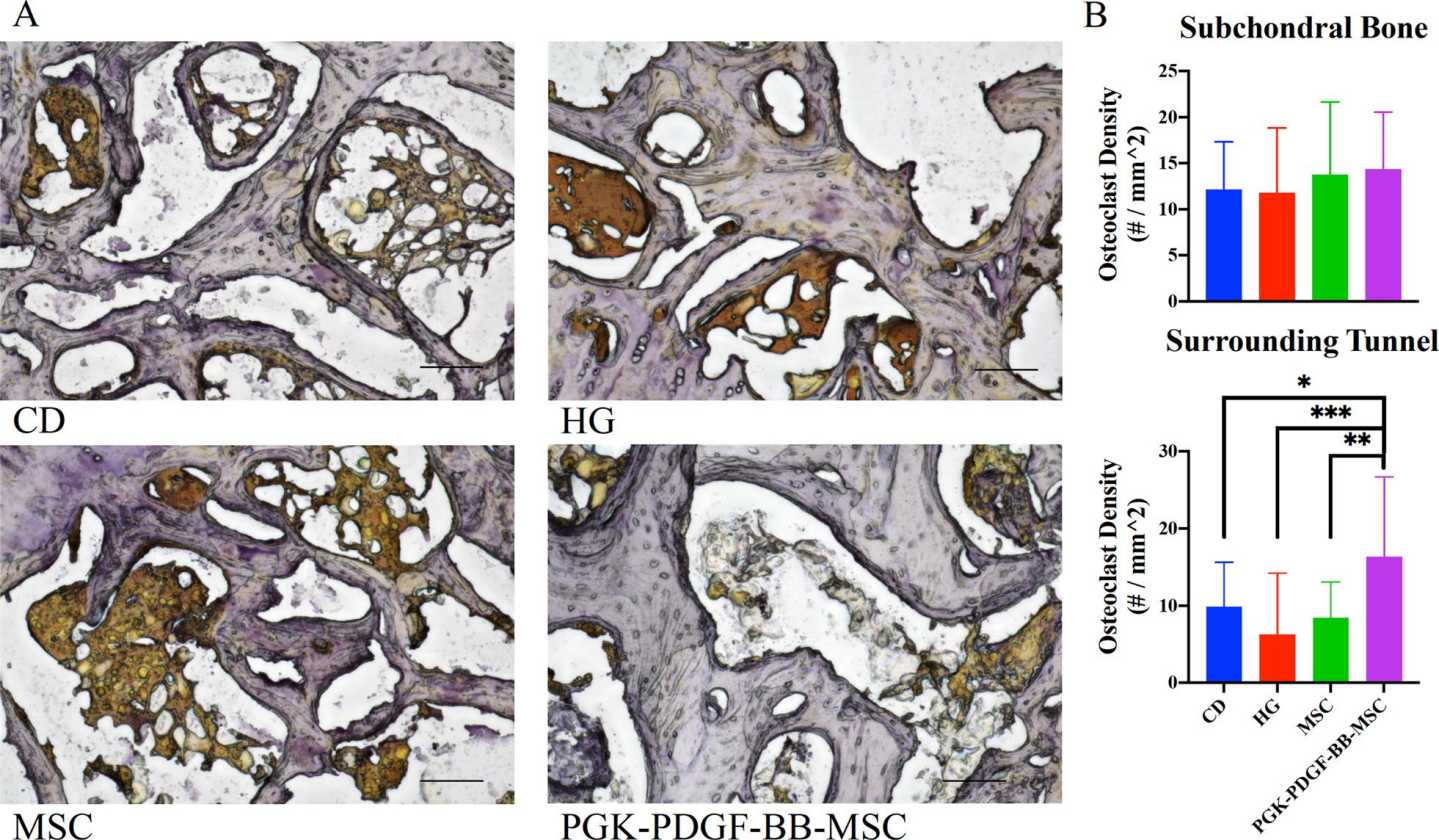
Assessment of osteoclastogenesis. TRAP staining was performed to evaluate osteoclast presence within the femoral head. **a** Representative images of each treatment group are shown. **b** Within the subchondral bone, no differences were noted between treatment groups. Within the bone surrounding the bone tunnel area, the PGK-PDGF-BB-MSC group demonstrated a significantly greater osteoclast density than the other treatment groups (*P* < 0.05 for all). Scale bar: 100 µm. *TRAP* tartrate-resistant acid phosphatase, *CD* core decompression, *HG* hydrogel, *MSC* mesenchymal stromal cell, *PGK-PDGF-BB-MSC* genetically modified phosphoglycerate kinase promoter platelet-derived growth factor-BB over-expressing MSC. *P < 0.05, **P < 0.01, ***P < 0.001

Representative images of the TRAP staining in the femoral head are shown in Fig. 7. The ICC for the two investigators counting the number of osteoclasts was *r* = 0.85 (95% confidence interval: 0.81–0.89, *P* < 0.0001). Results of the analysis are shown in Table 2. Within the subchondral bone, the osteoclast density of the PGK-PDGF-BB-MSC group was not significantly different than that of the CD, the HG, nor the MSC group. However, when assessing the area surrounding the bone tunnel, the PGK-PDGF-BB-MSC group demonstrated a significantly greater osteoclast density than the CD (*P* < 0.05), the HG (*P* < 0.001) and the MSC group (*P* < 0.01). No differences were detected between any of the other treatment groups.

**Table 2:** Results of the evaluation of osteoclastogenesis

Average osteoclast density (#/mm^2^) by position in femoral head (SD)						
	Subchondral bone					
	1	2	3	4	Total	*P* value
CD	13.9 (5.9)	14.0 (4.4)	10.2 (1.9)	10.4 (7.2)	12.1 (5.2)	0.361
HG	16.4 (9.5)	10.0 (5.5)	11.7 (3.0)	9.2 (7.7)	11.8 (7.0)	0.298
MSC	15.0 (6.4)	14.8 (7.0)	16.2 (12.4)	9.1 (2.7)	13.8 (7.9)	0.816
PGK-PDGF-BB-MSC	13.7 (3.8)	14.2 (6.6)	16.5 (7.7)	12.9 (7.3)	14.3 (6.2)	1.0

	Surrounding tunnel				
	5	6	7	Total	*P* value
CD	10.7 (4.1)	5.6 (4.8)	13.4 (6.0)	9.8 (5.8)	< 0.05
HG	9.9 (12.5)	4.9 (4.8)	4.0 (3.0)	6.3 (7.9)	< 0.001
MSC	10.6 (4.5)	6.9 (4.1)	7.8 (5.3)	8.4 (4.7)	< 0.01
PGK-PDGF-BB-MSC	17.5 (8.2)	13.7 (8.6)	17.8 (14.6)	16.4 (10.4)	1.0

The average osteoclast density for each position in the femoral head as described in Fig. 1 was calculated for each treatment group. The average osteoclast density for each region in the femoral head was calculated for each treatment group. A mixed effects model, where treatment group and femoral head position served as fixed effects, was used to perform the statistical analysis. The *P* values shown correspond to comparisons with the PGK-PDGF-BB-MSC treatment group. Comparisons between all other treatment groups did not reach statistical significance. *SD* standard deviation, *CD* core decompression, *HG* hydrogel, *MSC* mesenchymal stromal cell, *PGK-PDGF-BB-MSC* genetically modified phosphoglycerate kinase promoter platelet-derived growth factor-BB over-expressing MSC

#### Angiogenesis is enhanced throughout the femoral head by PDGF-BB-overexpressing MSCs

**Fig. 8:**
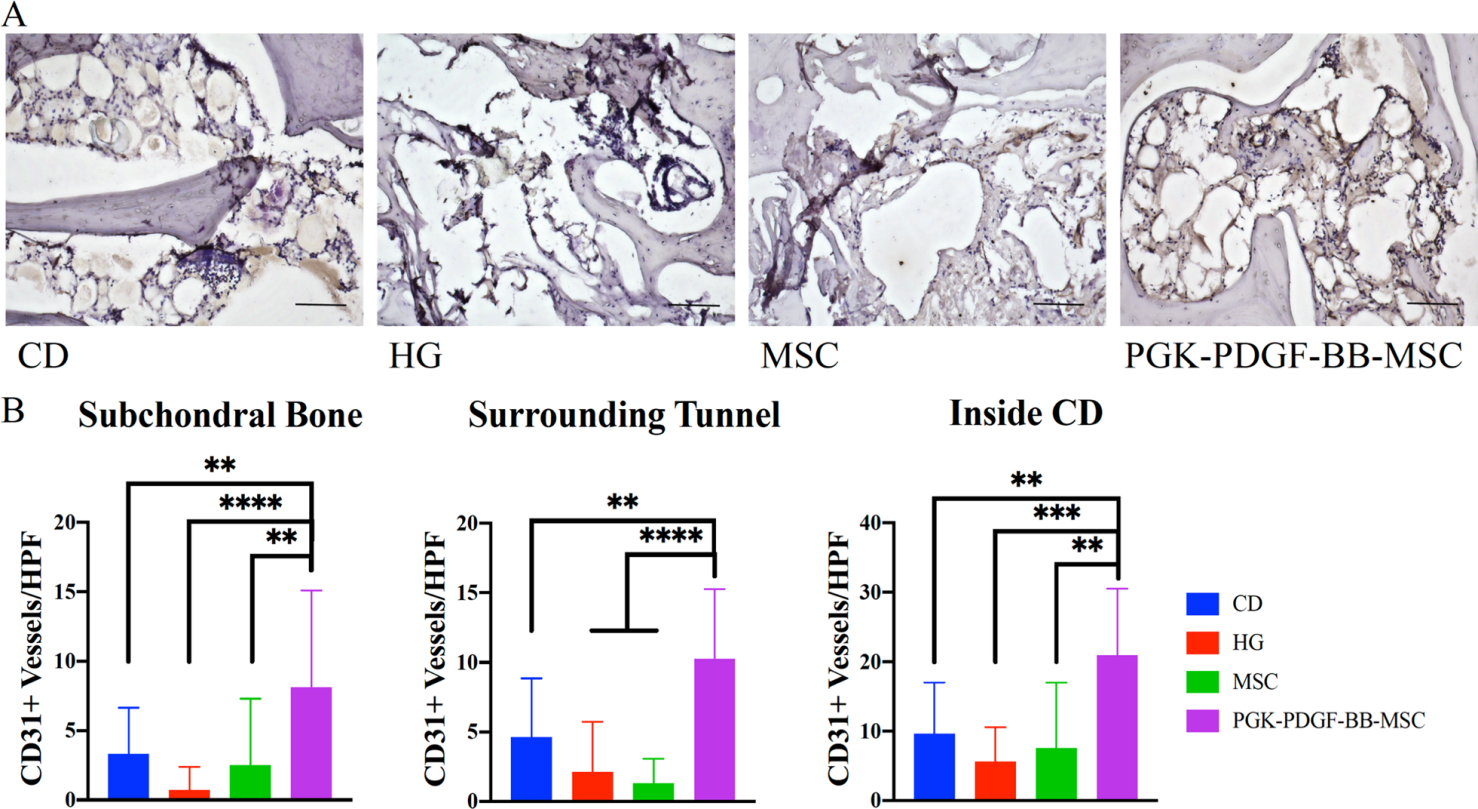
Assessment of angiogenesis. Immunohistochemistry was performed to stain for CD31+ microvessels within the femoral head. **a** Representative images of each treatment group are shown. **b** Within the subchondral bone, the bone surrounding the bone tunnel area, and within the bone tunnel area, the PGK-PDGF-BB-MSC group demonstrated a significantly greater number of CD31+ microvessels than the other treatment groups (*P* < 0.01 for all). Scale bar: 100 µm. CD31, cluster of differentiation 31; *CD* core decompression, *HG* hydrogel, *MSC* mesenchymal stromal cell, *PGK-PDGF-BB-MSC* genetically modified phosphoglycerate kinase promoter platelet-derived growth factor-BB over-expressing MSC. **P < 0.01, ***P < 0.001, ****P < 0.0001

Representative images of the CD31+ microvessel staining in the femoral head are shown in Fig. 8. The ICC for the two investigators counting the number of CD31+ microvessels was *r* = 0.93 (95% confidence interval: 0.91–0.95, *P* < 0.0001). The results of the analysis are shown in Table 3. When evaluating the high-power fields (HPF) within the subchondral bone, the PGK-PDGF-BB-MSC group had significantly greater microvessel formation than the CD (*P* < 0.01), the HG (*P* < 0.0001), and the MSC group (*P* < 0.01). Similarly, in the area surrounding the bone tunnel, the PGK-PDGF-BB-MSC group had significantly greater microvessel formation than the CD (*P* < 0.01), the HG (*P* < 0.0001), and the MSC group (*P* < 0.0001). Moreover, with regards to angiogenesis within the bone tunnel, the PGK-PDGF-BB-MSC group demonstrated significantly greater microvessel formation than the CD (*P* < 0.01), the HG (*P* < 0.001), and the MSC group (*P* < 0.01).

**Table 3:** Results of the evaluation of angiogenesis

Average number of CD31+ microvessels by position in femoral head (SD)						
	Subchondral bone					
	1	2	3	4	Total	*P* value
CD	2.0 (3.2)	5.5 (4.0)	2.0 (2.5)	3.8 (3.1)	3.3 (3.3)	< 0.01
HG	0.4 (0.7)	0.3 (0.6)	1.6 (3.2)	0.5 (1.0)	0.7 (1.7)	< 0.0001
MSC	2.3 (5.0)	4.3 (7.5)	2.2 (3.7)	1.3 (2.4)	2.5 (4.8)	< 0.01
PGK-PDGF-BB-MSC	10.5 (10.6)	6.3 (4.7)	9.4 (6.6)	6.3 (5.3)	8.1 (7.0)	1.0

	Surrounding tunnel				
	5	6	7	Total	*P* value
CD	1.6 (2.3)	5.2 (4.3)	7.1 (4.4)	4.6 (4.2)	< 0.01
HG	0.6 (1.2)	1.7 (2.9)	3.6 (5.4)	2.1 (3.6)	< 0.0001
MSC	0.3 (0.4)	1.7 (1.7)	1.9 (2.4)	1.3 (1.8)	< 0.0001
PGK-PDGF-BB-MSC	8.4 (3.6)	11.0 (6.2)	11.3 (5.4)	10.3 (5.0)	1.0

	Inside CD				
	8	9	10	Total	*P* value
CD	10.0 (8.5)	10.3 (6.6)	8.5 (8.2)	9.6 (7.4)	< 0.01
HG	6.0 (5.2)	5.7 (5.4)	5.1 (5.3)	5.6 (5.0)	< 0.001
MSC	4.6 (4.6)	7.9 (8.9)	10.2 (13.5)	7.6 (9.4)	< 0.01
PGK-PDGF-BB-MSC	16.5 (6.8)	20.4 (8.5)	25.9 (11.8)	20.9 (9.6)	1.0

The average number of CD31+  microvessels for each position in the femoral head as described in Fig. 1 was calculated for each treatment group. The average number of CD31+ microvessels for each region in the femoral head was calculated for each treatment group. A mixed effects model, where treatment group and femoral head position served as fixed effects, was used to perform the statistical analysis. The *P* values shown correspond to comparisons with the PGK-PDGF-BB-MSC treatment group. Comparisons between all other treatment groups did not reach statistical significance. *SD* standard deviation, *CD* core decompression, *HG* hydrogel, *MSC* mesenchymal stromal cell, *PGK-PDGF-BB-MSC* genetically modified phosphoglycerate kinase promoter platelet-derived growth factor-BB over-expressing MSC

## Discussion

This study sought to assess the proliferative and osteogenic potential of PDGF-BB-MSCs in vitro and to evaluate their therapeutic effect as an adjunctive treatment of CD in an in vivo rabbit model of steroid-associated ONFH. We found that MSCs that overexpress PDGF-BB had greater proliferative and osteogenic differentiation potential than unmodified MSCs. Additionally, the use of these genetically modified MSCs as an augmentative therapy leads to improved bone mineralization, decreased osteonecrotic lesions, increased osteoclastogenesis, and enhanced angiogenesis.

A wide array of promoters has been used for gene expression in various cell types transduced with lentiviral vectors [27–29]. Specifically, the CMV and PGK promoters have been previously utilized to stimulate gene expression in genetically modified MSCs [16, 30, 31]. Interestingly, prior studies have noted that the CMV promoter activity may be greater or lower than the PGK depending on the type of cell that is transduced [31–33]. These discrepancies may be due to the epigenetic modifications that are made to the CMV promoter post-transduction, which lead to variable amounts of gene silencing [34, 35]. Here, we found that the CMV promoter led to significantly greater expression of PDGF-BB than the PGK promoter; however, this did not translate to enhanced cellular function. Our results suggest that a low, rather than a high level of PDGF-BB expression leads to greater acceleration of cellular replication. Furthermore, we found a similar pattern when evaluating the osteogenic potential of these engineered cells. The latter result is supported by a previous in vitro investigation that demonstrated increasing mineralization with PDGF-BB concentrations of 0.3 and 3.0 ng/ml, but minimal mineralization when 30 ng/ml were used [11]. These results suggest that lower concentrations of PDGF-BB initially have a stimulatory effect on MSCs, but higher concentrations may cause PDGF-BB to have an inhibitory effect on MSCs. Additionally, the observed increase in ALP staining without a reciprocal increase in Alizarin Red staining for the genetically modified cells may suggest that PDGF-BB’s stimulatory effect may be strongest in the early stages of osteogenesis. Prior in vitro studies have also demonstrated that EPC migration and tube formation are stimulated after activation of the PI3K/Akt pathway by PDGF-BB signaling, which has established the role of PDGF-BB in angiogenesis [36]. Lastly, osteoclastogenesis has also been shown to be enhanced in vitro by PDGF-BB signaling [37]. Given the findings of our in vitro experiments, coupled with the results of other studies, we determined that the use of PGK-PDGF-BB-MSCs would be best suited for the in vivo portion of our study.

In our in vivo rabbit model of steroid-associated ONFH, injection of PGK-PDGF-BB-MSCs at the time of CD demonstrated a trend towards greater BMD in the area of the femoral head outside of the bone tunnel, compared to the CD-only treatment. Given that bone mineralization is a process that involves a primary stage that may last several months and a secondary stage that may last several years, it is possible that our findings reflect only the initial divergence in bone mineralization capacity between treatment groups [38, 39]. Therefore, a timepoint of greater than eight weeks may have allowed for better detection of significant differences in BMD between treatment groups. Interestingly, we did not see a similar trend with regard to BVF. However, it is possible that this latter finding may have been influenced by enhanced bone resorption in the PGK-PDGF-BB-MSC group given its enhanced osteoclastogenesis. Indeed, the demonstration that PDGF-BB can stimulate osteoclast formation and maturation in this study parallels the findings of others [10, 12–14]. Although the ability of osteoclasts to resorb and remodel bone is essential for skeletal homeostasis, osteoclastic activity is linked to and balanced with the function of osteoprogenitors to maintain bone homeostasis [40–42]. This balance may also translate to the physical properties of bone and, as our mechanical testing results suggest, augmentation of CD with cell-based therapy may be insufficient to enhance this property. Therefore, the addition of an osteoinductive scaffold may be necessary to improve the mechanical properties of bone in the treatment of early-stage ONFH [21].

The exact pathophysiology of ONFH is yet to be described; however, glucocorticoid use is a well-established risk factor for its development [43–47]. Histologically, diffuse bone marrow damage and osteocyte death is a common feature of steroid-associated ONFH [48, 49]. Moreover, in early-stage ONFH, such as Association Research Circulation Osseous (ARCO) stages 1 and 2 collapse of the femoral head has not yet taken place [50]. Therefore, given that disease progression cannot be determined on the basis of X-ray findings in the stages where CD is the most common treatment modality, histologic evaluation of osteonecrotic lesions is paramount to assessment of potential treatments [50]. We qualitatively confirmed that osteonecrosis developed in the femoral head of all rabbits, as each specimen contained changes in bone marrow reflective of this degenerative process, such as the presence of enlarged fat cells and granulation tissue within marrow spaces [19, 24]. Furthermore, apoptotic changes in osteocytes such as shrunken and eccentric nuclei were also observed in all specimens [51, 52]. To perform a quantitative evaluation, we compared the percentage of empty lacunae, an established sign of osteonecrosis, between treatment groups [3]. We found that augmentation of CD with PGK-PDGF-BB-MSCs was associated with a lower percentage of empty lacunae than all other treatment groups. Apoptosis in steroid-associated ONFH develops by upregulated expression of caspase-3 via activation of signal transducer and activator of transcription 1 (STAT-1) [53]. PDGF-BB leads to a reduction in caspase-3 cleavage in cells such as chondrocytes, cardiomyocytes, and neurons after subjecting them to inflammatory conditions [15, 54, 55]. Additionally, PDGF-BB also increases the expression of the anti-apoptotic protein, Bcl-2, in intervertebral disc cells [56]. Together, these reports highlight PDGF-BB’s ability to function as an anti-inflammatory molecule and may explain why we observed a reduction in the osteonecrotic lesions in our model of steroid-associated ONFH.

Vascular damage is a frequently cited issue in the pathogenesis of ONFH [1, 2, 4, 47, 48, 57]. Enhancement of angiogenesis had a regenerative and healing effect in other animal models of steroid-associated ONFH [58, 59]. Moreover, PDGF-BB is known to increase EPC migration and promote angiogenesis [10, 12–14, 36]. We found that the addition of PGK-PDGF-BB-MSCs to CD led to greater angiogenesis within the bone tunnel and in the surrounding femoral head. This pro-angiogenic effect may have been a contributing factor in the reduction in the prevalence of the osteonecrotic lesions observed.

Interestingly, all of our histological analyses demonstrated that the effect of PGK-PDGF-BB-MSCs was more pronounced in the area of bone surrounding the bone tunnel than in the subchondral bone. This localized effect suggests that PDGF-BB signaling may preferentially occur in a paracrine fashion. In fact, paracrine effects of PDGF-BB have been reported previously, in different tissue types [60–62]. This observation may have important implications for PDGF-BB-based treatments of early-stage ONFH as these may need to be planned in a spatially aware manner so as to optimize the effect and benefit of the growth factor.

Several limitations must be considered when interpreting the results of our study. First, our conclusions may not be directly translatable to human disease as we used a rabbit model of ONFH. Microanatomical analyses of rabbit hips have shown that they are somewhat different from humans, which may have consequences with regards to treatment outcomes between species [63]. Furthermore, as this is a model of early-stage ONFH, femoral head collapse prior to the time of the CD procedure would not be present radiographically. Prior studies have demonstrated that magnetic resonance imaging can detect changes six weeks after steroid injection at the earliest, despite there being histologic changes in the femoral head by this time [64, 65]. However, this protocol successfully induced ONFH in rabbits in prior investigations in a similar timeframe [20, 66, 67]. The HG used to suspend the MSCs has been demonstrated to be a suitable environment for cell replication for at least 28 days after suspension [18]. However, the viability of the unmodified MSCs and PGK-PDGF-BB-MSCs after injection into the bone tunnel must also be verified. A prior murine study demonstrated that injected MSCs are initially viable, yet more than 90% of them may be lost by four weeks post-injection [17]. Therefore, our results may reflect the effect of PDGF-BB in the first few days to weeks after injection. Lastly, our study utilized an intervention that requires genetic modification in order to facilitate osteogenesis and angiogenesis, and to serve as a chemotactic factor for other immune cells that assist in these processes [68]. However, as of 2017, many clinical trials investigating gene therapies have taken place, 196 of which specifically used lentiviral vectors for gene modification [69]. Future work would need to establish the safety profile, as well as the efficacy and cost-effectiveness of an intervention like the one used in this study.

## Conclusions

In conclusion, PDGF-BB over-expressing MSCs are able to accelerate cellular proliferation and osteogenic differentiation in vitro. Additionally, augmentation of CD with PGK-PDGF-BB-MSCs led to greater BMD, osteoclastogenesis, angiogenesis, and decreased histological findings of osteonecrosis in an in vivo rabbit model of steroid-associated ONFH. These findings suggest that augmentation of CD with PDGF-BB-MSCs may allow for greater bone healing and regeneration during treatment of early-stage ONFH.

## Data Availability

All of the data are included in the paper.

## Abbreviations

ONFH: Osteonecrosis of the femoral head
OA: Osteoarthritis
THA: Total hip arthroplasty
CD: Core decompression
PDGF: Platelet-derived growth factor
MSCs: Mesenchymal stromal cells
EPCs: Endothelial progenitor cells
BRONJ: Bisphosphonate-related osteonecrosis of the jaw
IL-1β: Interleukin-1-beta
NF-κB: Nuclear factor kappa B
PDGF-BB-MSCs: PDGF-BB-overexpressing MSCs
GM: Growth medium
α-MEM: α-Minimal Essential Medium
FBS: Fetal bovine serum
CMV: Cytomegalovirus
PGK: Phosphoglycerate kinase
MOI: Multiplicity of infection
CMV-PDGF-BB-MSCs: PDGF-BB-MSCs under control of the CMV promoter
PGK-PDGF-BB-MSCs: PDGF-BB-MSCs under control of the PGK promoter
PBS: Phosphate buffered saline
ALP: Alkaline phosphatase
OM: Osteogenic medium
PFA: Paraformaldehyde
HG: Hydrogel
DI: Deionized
BMD: Bone mineral density
BVF: Bone volume fraction
ROI: Region of interest
TV: Total volume
BV: Bone volume
BMC: Bone mineral content
PMMA: Polymethylmethacrylate
EDTA: Ethylenediaminetetraacetic acid
OCT: Optimal cutting temperature
H&E: Hematoxylin and eosin
TRAP: Tartrate-resistant acid phosphatase
ICC: Intraclass correlation coefficients
ARCO: Association research circulation osseous
STAT-1: Signal transducer and activator of transcription 1

## References

[1] Mont MA (2020). Nontraumatic osteonecrosis of the femoral head: Where do we stand today? A 5-year update. J Bone Joint Surg Am.

[2] Koo K-H, et al. Osteonecrosis. 2016.

[3] Mankin HJ (1992). Nontraumatic necrosis of bone (osteonecrosis). N Engl J Med.

[4] Moya-Angeler J (2015). Current concepts on osteonecrosis of the femoral head. World J Orthop.

[5] Goodman SB, Maruyama M (2020). Inflammation, bone healing and osteonecrosis: from bedside to bench. J Inflamm Res.

[6] Musso ES (1986). Results of conservative management of osteonecrosis of the femoral head. A retrospective review. Clin Orthop Relat Res.

[7] Hua KC (2019). The efficacy and safety of core decompression for the treatment of femoral head necrosis: a systematic review and meta-analysis. J Orthop Surg Res.

[8] Maruyama M (2019). Cell-based and scaffold-based therapies for joint preservation in early-stage osteonecrosis of the femoral head: a review of basic research. JBJS Rev.

[9] Majidinia M, Sadeghpour A, Yousefi B (2018). The roles of signaling pathways in bone repair and regeneration. J Cell Physiol.

[10] Xie H (2014). PDGF-BB secreted by preosteoclasts induces angiogenesis during coupling with osteogenesis. Nat Med.

[11] Chen W (2015). PDGFB-based stem cell gene therapy increases bone strength in the mouse. Proc Natl Acad Sci USA.

[12] Su W (2020). Angiogenesis stimulated by elevated PDGF-BB in subchondral bone contributes to osteoarthritis development. JCI Insight.

[13] Gao SY (2017). Zoledronate suppressed angiogenesis and osteogenesis by inhibiting osteoclasts formation and secretion of PDGF-BB. PLoS ONE.

[14] Gao SY (2021). PDGF-BB exhibited therapeutic effects on rat model of bisphosphonate-related osteonecrosis of the jaw by enhancing angiogenesis and osteogenesis. Bone.

[15] Montaseri A (2011). IGF-1 and PDGF-bb suppress IL-1beta-induced cartilage degradation through down-regulation of NF-kappaB signaling: Involvement of Src/PI-3K/AKT pathway. PLoS ONE.

[16] Zhang N (2021). PDGF-BB and IL-4 co-overexpression is a potential strategy to enhance mesenchymal stem cell-based bone regeneration. Stem Cell Res Ther.

[17] Lin T (2018). Transplanted interleukin-4–secreting mesenchymal stromal cells show extended survival and increased bone mineral density in the murine femur. Cytotherapy.

[18] Moeinzadeh S (2021). In-situ stable injectable collagen-based hydrogels for cell and growth factor delivery. Materialia.

[19] Yamamoto T (1997). Effects of pulse methylprednisolone on bone and marrow tissues: corticosteroid-induced osteonecrosis in rabbits. Arthritis Rheum.

[20] Motomura G (2008). Dose effects of corticosteroids on the development of osteonecrosis in rabbits. J Rheumatol.

[21] Maruyama M (2018). The effects of a functionally-graded scaffold and bone marrow-derived mononuclear cells on steroid-induced femoral head osteonecrosis. Biomaterials.

[22] Maruyama M (2020). The efficacy of core decompression for steroid-associated osteonecrosis of the femoral head in rabbits. J Orthop Res.

[23] Bergmann G (2016). Standardized loads acting in hip implants. PLoS ONE.

[24] Kawai K, Tamaki A, Hirohata K (1985). Steroid-induced accumulation of lipid in the osteocytes of the rabbit femoral head. A histochemical and electron microscopic study. J Bone Joint Surg Am.

[25] Ren P-G (2008). Quantitation of bone area in undecalcified frozen sections with fluorescent microscopy. J Histotechnol.

[26] Maruyama M (2021). The efficacy of lapine preconditioned or genetically modified IL4 over-expressing bone marrow-derived mesenchymal stromal cells in corticosteroid-associated osteonecrosis of the femoral head in rabbits. Biomaterials.

[27] Li M (2010). Optimal promoter usage for lentiviral vector-mediated transduction of cultured central nervous system cells. J Neurosci Methods.

[28] Soda YK, Li X, Bai Y, Cho SG, Futami M, Chen M, Kobayashi S, Miyoshi H, Sumimoto H, Ohga S, Hara T, Tojo A, Asano S (2005). PGK and CMV promoters exert the strongest activity in lentiviral gene transduction of myeloid cells including mature neutrophils. Mol Ther.

[29] Ramezani A, Hawley TS, Hawley RG (2000). Lentiviral vectors for enhanced gene expression in human hematopoietic cells. Mol Ther.

[30] McGinley L (2011). Lentiviral vector mediated modification of mesenchymal stem cells & enhanced survival in an in vitro model of ischaemia. Stem Cell Res Ther.

[31] Qin JY (2010). Systematic comparison of constitutive promoters and the doxycycline-inducible promoter. PLoS ONE.

[32] Norrman K (2010). Quantitative comparison of constitutive promoters in human ES cells. PLoS ONE.

[33] Nieuwenhuis B (2021). Optimization of adeno-associated viral vector-mediated transduction of the corticospinal tract: comparison of four promoters. Gene Ther.

[34] Teschendorf C (2002). Comparison of the EF-1 alpha and the CMV promoter for engineering stable tumor cell lines using recombinant adeno-associated virus. Anticancer Res.

[35] Brooks AR (2004). Transcriptional silencing is associated with extensive methylation of the CMV promoter following adenoviral gene delivery to muscle. J Gene Med.

[36] Wang H (2012). Over-expression of PDGFR-beta promotes PDGF-induced proliferation, migration, and angiogenesis of EPCs through PI3K/Akt signaling pathway. PLoS ONE.

[37] Li DQ (2017). Platelet-derived growth factor BB enhances osteoclast formation and osteoclast precursor cell chemotaxis. J Bone Miner Metab.

[38] Boivin G, Meunier PJ (2003). Methodological considerations in measurement of bone mineral content. Osteoporos Int.

[39] Hernandez CJ (2001). A theoretical analysis of the contributions of remodeling space, mineralization, and bone balance to changes in bone mineral density during alendronate treatment. Bone.

[40] Rowe P, Koller A, Sharma S. Physiology, bone remodeling. In: StatPearls, Treasure Island (FL); 2020. 29763038

[41] Foger-Samwald U (2020). Osteoporosis: pathophysiology and therapeutic options. EXCLI J.

[42] Bailey JR, Tapscott DC. Osteopetrosis. In: StatPearls, Treasure Island (FL); 2020.

[43] Mont MA (2015). High-dose corticosteroid use and risk of hip osteonecrosis: meta-analysis and systematic literature review. J Arthroplasty.

[44] Fukushima W (2010). Nationwide epidemiologic survey of idiopathic osteonecrosis of the femoral head. Clin Orthop Relat Res.

[45] Zhang NF (2008). Steroid-induced osteonecrosis: the number of lesions is related to the dosage. J Bone Joint Surg Br.

[46] Kubo T (2016). Clinical and basic research on steroid-induced osteonecrosis of the femoral head in Japan. J Orthop Sci.

[47] Petek D, Hannouche D, Suva D (2019). Osteonecrosis of the femoral head: pathophysiology and current concepts of treatment. EFORT Open Rev.

[48] Wang A, Ren M, Wang J (2018). The pathogenesis of steroid-induced osteonecrosis of the femoral head: a systematic review of the literature. Gene.

[49] Wei Q (2018). Microarchitecture features and pathology of necrotic region in patients with steroid-induced and alcohol-induced osteonecrosis of femoral head. Zhongguo Xiu Fu Chong Jian Wai Ke Za Zhi.

[50] Larson E (2018). Early-stage osteonecrosis of the femoral head: Where are we and where are we going in year 2018?. Int Orthop.

[51] Usui Y, Kawai K, Hirohata K (1989). An electron microscopic study of the changes observed in osteocytes under ischemic conditions. J Orthop Res.

[52] Weinstein RS, Nicholas RW, Manolagas SC (2000). Apoptosis of osteocytes in glucocorticoid-induced osteonecrosis of the hip. J Clin Endocrinol Metab.

[53] Xu X (2014). STAT1-caspase 3 pathway in the apoptotic process associated with steroid-induced necrosis of the femoral head. J Mol Histol.

[54] Hsieh PC (2006). Controlled delivery of PDGF-BB for myocardial protection using injectable self-assembling peptide nanofibers. J Clin Invest.

[55] Zheng L (2010). Neuroprotective effects of PDGF against oxidative stress and the signaling pathway involved. J Neurosci Res.

[56] Presciutti SM (2014). PDGF-BB inhibits intervertebral disc cell apoptosis in vitro. J Orthop Res.

[57] Saito S, Ohzono K, Ono K (1992). Early arteriopathy and postulated pathogenesis of osteonecrosis of the femoral head. The intracapital arterioles. Clin Orthop Relat Res.

[58] Li J (2015). The effect of deferoxamine on angiogenesis and bone repair in steroid-induced osteonecrosis of rabbit femoral heads. Exp Biol Med.

[59] Xu T (2017). Administration of erythropoietin prevents bone loss in osteonecrosis of the femoral head in mice. Mol Med Rep.

[60] Xue Y (2011). PDGF-BB modulates hematopoiesis and tumor angiogenesis by inducing erythropoietin production in stromal cells. Nat Med.

[61] deJong JS (1998). Expression of growth factors, growth inhibiting factors, and their receptors in invasive breast cancer I: an inventory in search of autocrine and paracrine loops. J Pathol.

[62] Battegay EJ (1994). PDGF-BB modulates endothelial proliferation and angiogenesis in vitro via PDGF beta-receptors. J Cell Biol.

[63] Bagi CM, Berryman E, Moalli MR (2011). Comparative bone anatomy of commonly used laboratory animals: implications for drug discovery. Comp Med.

[64] Kubo T (1997). Initial MRI findings of non-traumatic osteonecrosis of the femoral head in renal allograft recipients. Magn Reson Imaging.

[65] Fujioka M (2001). Initial changes of non-traumatic osteonecrosis of femoral head in fat suppression images: bone marrow edema was not found before the appearance of band patterns. Magn Reson Imaging.

[66] Zhao G (2013). Cholesterol- and lanolin-rich diets may protect against steroid-induced osteonecrosis in rabbits. Acta Orthop.

[67] Kang P (2015). Repairing defect and preventing collapse of femoral head in a steroid-induced osteonecrotic of femoral head animal model using strontium-doped calcium polyphosphate combined BM-MNCs. J Mater Sci Mater Med.

[68] Pierce GF (1989). Platelet-derived growth factor and transforming growth factor-beta enhance tissue repair activities by unique mechanisms. J Cell Biol.

[69] Ginn SL (2018). Gene therapy clinical trials worldwide to 2017: an update. J Gene Med.

